# Anti-miRNA- 155 - 5p enhances the cytotoxic efficacy of temozolomide in brain cancer stem cells

**DOI:** 10.1007/s00210-025-04135-6

**Published:** 2025-04-11

**Authors:** Lütfiye Ozpak, Bakiye Göker Bağca, Çiğir Biray Avci, Cumhur Gündüz, Burak Durmaz

**Affiliations:** 1 Department of Medical Biology, Faculty of Medicine, Sütçü İMam University, Kahramanmaraş, Turkey; 2 Department of Medical Biology, Faculty of Medicine, Aydın Adnan Menderes University, Aydın, Turkey; 3 Department of Medical Biology, Faculty of Medicine, Ege University, İzmir, Turkey; 4 Department of Medical Genetics, Faculty of Medicine, Ege University, İzmir, Turkey

**Keywords:** MiRNA, MiR- 155 - 5p, Brain cancer stem cell, Temozolomide resistance, Glioblastoma

## Abstract

This study aimed to assess the molecular effects of the miR- 155 - 5p in modulating Temozolomide chemosensitivity, which was examined utilizing in silico and in vitro methods on brain cancer stem cells (BCSCs) that exhibit high differentiation, apoptosis avoidance, and a remarkable capacity for self-renewal as a mechanism of chemotherapeutic resistance. The hypothesis of the study is how miR- 155 - 5p modulates temozolomide chemosensitivity in brain cancer stem cells (BCSCs) and their differentiated counterparts (BSCs) and whether it influences the molecular mechanisms underlying tumour recurrence and treatment failure. By silencing miR- 155 - 5p using anti-miR oligonucleotides, the knockdown efficiency of miR- 155 - 5p was confirmed via quantitative qRT-PCR. The increased chemosensitivity effect of miR- 155 - 5p against temozolomide in BCSCs and BCSs was measured by cytotoxicity assay. Subsequently, all studied groups were evaluated by performing cell viability, cell cycle, and apoptosis tests. miRNA-mRNA-pathway interactions were analyzed using different bioinformatics tools and databases. Cell viability was significantly reduced by 42.9% in the combined treatment (anti-miR- 155 - 5p + temozolomide) group compared to the untreated group. Temozolomide treatment in combination with anti-miR- 155 - 5p showed a significant decrease in cell viability compared to temozolomide alone. In contrast, late apoptosis increased 2.10-fold (*p* < 0.0001), indicating a synergistic effect in promoting late apoptosis. Combining temozolomide and anti-miR- 155 - 5p inhibits DNA synthesis by causing G2 phase arrest. mRNAs targeted by miR- 155 - 5p and significantly down-regulated in glioblastoma included *GABRA1*, *GABRB2*, *SCN1A*, *GRIN2A*, and *SGIP1*. By survival analysis, low expression of *SCN1A* was associated with poor prognosis (*p* < 0.05; HR = 0.7), highlighting its potential prognostic role. The combination of temozolomide treatment with suppression of miR- 155 - 5p may provide a more effective and side-effect minimized brain cancer treatment strategy by reducing resistance.

**Supplementary Information:**

The online version contains supplementary material available at 10.1007/s00210-025-04135-6.

## Introduction

Glioblastoma is the most pernicious type of tumor originating from the nervous system, is aggressive, and is associated with a poor prognosis (Kanderi and Gupta 2022; Stupp et al. 2019). Because of its high mortality, low survival rates, economic burden, and impact on negative quality of life, it is an important part of the global burden of disease. With the most recent World Health Organization (WHO) classification, an integrated diagnostic strategy based on molecular markers has become more significant in diagnosing and prognosis of gliomas (Melhem et al. 2022). Glioblastoma displays intra- and inter-tumor variability and heterogeneity, which makes an individualized medical approach difficult. In glioblastoma samples, BCSCs found in angiogenic regions belong to a subpopulation of cells with self-renewal, proliferation, and tumor propagation capabilities, attributed to increased angiogenesis and drug resistance (Chen et al. 2012). Cancer stem cells are thought to be the cause of tumor recurrence and therapy failure. They also re-initiate cancer growth and resist existing conventional treatment methods. As a result, knowing the molecular mechanism underlying BCSC-mediated tumor recurrence becomes crucial for therapeutic strategy (Yu et al. 2016). At present, standard treatment involves removal of the tumor, radiotherapy, and chemotherapy with temozolomide. However, due to the invasive nature of the tumor, complete surgical removal is not always successful, which may cause the remaining tumor cells to grow and lead to recurrences (Herbener et al 2020; Dhungel et al 2024).

Temozolomide is a classical chemotherapeutic agent used for the management of glioblastoma and received Food and Drug Administration (FDA) approval in 2005. The high development of resistance to temozolomide is associated with mechanisms such as DNA repair (MGMT), drug delivery, and survival autophagy. Therefore, it is necessary to develop new approaches, such as effective drug delivery systems and different uses of therapeutic drugs, which can potentially increase survival (Karachi et al. 2018; Jiapaer et al. 2018).

miRNAs are short, single-stranded, non-coding types of RNA of epigenetic bioregulation that enable mRNA degradation by sequence-specific base pairing to the 3′ UTR of targeted mRNAs. The latest research highlights miRNAs as promising biomarkers in the diagnosis and prognosis of glioblastoma and the regulation of mechanisms contributing to radiotherapy and chemotherapy resistance (Valle-Garcia et al. 2024; Ahmed et al 2021). Dysregulated expression of certain microRNAs has been reported to trigger multi-drug resistance (MDR) in glioblastoma by impairing the function of apoptotic pathways and promoting uncontrolled proliferation. Suppression of miR- 497 in GB cells resulted in markedly increased apoptosis and increased sensitivity of these cells to temozolomide. Similarly, treatment by suppression of miR- 21 in glioblastoma cells increased apoptosis induction compared to temozolomide treatment alone, thus contributing to breaking drug resistance (Mahinfar et al. 2022; Wang et al. 2016). It has been pointed out that miR- 155 - 5p and miR‑221‑3p play an important role in developing MDR by targeting caspase- 3 in glioblastoma cells (Milani et al. 2019). Although miR- 155 is primarily recognized as an oncogenic miRNA in leukemia, recent findings on its role in breast cancer progression, including 147 target genes and their influence on biological pathways such as apoptosis, differentiation, angiogenesis, proliferation, and epithelial-mesenchymal transition, demonstrate the clinical and research potential of this miRNA (Delen and Doğanlar 2022).

In this study, we aimed to determine the role of miR- 155 - 5p in temozolomide chemosensitivity in BCSCs due to it has strong differentiating ability, avoids apoptosis, and marked capacity for self-renewal in chemotherapeutic resistance mechanisms. By silencing miR- 155 - 5p, apoptosis, proliferation, and in silico analysis, we attempted to determine whether miR- 155 - 5p has a function in the temozolomide cytotoxic efficacy phenotype in the BCSCs.

## Materials and methods

### Cell culture

Two distinct cell lines were utilized: human brain cancer stem cell and brain stem cell. BCSCs were obtained from Celprogen (Catalog No: 36110–37), and BSCs were sourced from the same provider (Catalog No: 36109–36, USA). According to the manufacturer's datasheets, BCSCs express several cancer stem cell markers (e.g., CD133, CD44, Oct4, ALDH), and BSCs express neural and glial lineage markers (e.g., Nestin, GFAP, MAP2, NF-L). As these characteristics have been validated by the manufacturer and listed in Supplementary Table 1, additional experimental verification of marker expression was not conducted in the scope of this study. Upon acquisition, BCSCs and BSCs were maintained via Human Brain Cancer Stem Cell Complete Growth Media (Celprogen, Cat. No: M36110 - 37) and Human Brain Stem Cell Media (Celprogen, Cat. No: M36109 - 36), respectively in the incubator with 37 °C in a humidified atmosphere with 5% CO_2_. Media were refreshed every 48 to 72 h, depending on cell confluency, and cells were subcultured once they reached 70–80% confluency. Cell viability was monitored via Trypan blue assay (Biological Industries, Israel, Cat. No: 03–102 - 1B).

### RNA interference and validation

Exploring the functional effects of miR- 155 - 5p, we utilized RNA interference to silence its expression using anti-miR oligonucleotides. Anti-miR- 155 - 5p (Thermo, Waltham, Massachusetts Cat. No: 483064) was employed for the knockdown experiments, with Silencer Select Negative Control (Thermo, Cat. No: 4390843) used as a negative control. Cells were seeded in 6-well plates at a density of 300,000 cells per well in 1,500 µL of culture medium and incubated for 24 h. Transfection was employed via Opti-MEM Reduced Serum Medium (Gibco, Thermo Fisher Scientific, Waltham, MA, USA, Cat. No: 11058–021) in conjunction with Lipofectamine 2000 (Thermo, Cat. No: 11668030) as the transfection reagent, following the manufacturer’s protocol to ensure efficient delivery and silencing. For each well, 30 pmol of siRNA and 9 µL of Lipofectamine 2000 were used to ensure efficient delivery and silencing. Cells were incubated for 24 h post-transfection before further analysis.

### qRT-PCR

Total RNA, including small RNA, was extracted from the cells using miRNeasy Mini Kit (Qiagen, Hilden, Germany, Cat. No: 217004) according to the manufacturer’s instructions. RNA quality and concentration were assessed using a Nabi (Microdigital) spectrophotometer. Following that, the miScript II RT Kit (Qiagen, Cat. No: 218160), which is optimized for small RNA templates, including miRNAs, was exploited for cDNA synthesis under standard thermal cycler conditions.

RT-qPCR analysis was conducted using SYBR Green Master Mix and primer pairs for miR- 155 - 5p and U6 small nuclear RNA as an internal control (Supplementary Table 2). Amplifications were performed on the CFX Maestro Instrument (Bio-Rad, Hercules, CA, USA). qPCR conditions were as follows: an initial activation step at 95 °C for 15 min to activate HotStarTaq DNA Polymerase, followed by 40 cycles of a three-step cycling process consisting of denaturation at 94 °C for 15 s, annealing at 55 °C for 30 s, and extension at 70 °C for 30 s, during which fluorescence data collection was performed. Relative miR- 155 - 5p expression was normalized to the endogenous control U6 and was utilized and calculated by the ∆∆Ct method. Each sample underwent three replicates.

For mRNA expression analysis, cDNA synthesis was performed using the WizScript™ cDNA Synthesis Kit (WizBio, Cat. No: W2201, Korea), following the manufacturer's protocol. Gene expression levels of apoptosis-related (*BAX*, *BCL2*), cell cycle-related (*CCND1*, *PCNA*), metastasis-related (*MMP2*, *MMP9*) and potential downstream targets of miR- 155 - 5p (*GABRA1*, *SCN1A*) genes were assessed by RT-qPCR using specific primers (Supplementary Table 2). GAPDH was used as the internal control for normalization. mRNA qPCR amplifications were also performed using the CFX Maestro Instrument (Bio-Rad, Hercules, CA, USA), following the same cycling conditions described above. Relative expression levels were calculated using the ∆∆Ct method, and each reaction was run in triplicate.

### Preparation of chemical agent

50 mM Temozolomide (Sigma-Aldrich, St. Louis, MO, USA Cat. No: T2577) stock was prepared via DMSO and aliquoted and stored at − 20 °C to maintain stability and minimize freeze–thaw cycles. To perform experiments, the stock was thawed and diluted to the desired working concentrations using the appropriate cell culture medium.

### Temozolomide cytotoxicity assay

A resazurin and resorufin-based cell viability was employed to determine the cytotoxic effects of temozolomide. BCSC cells were seeded at 5 × 10^4^ cells/mL a concentration into 96-well plates and incubated for 24 h. A temozolomide stock solution was diluted with culture media to achieve treatment concentrations of 0.1, 0.25, 0.5, 1, 1.5, and 2 mM, and the cells were exposed to these concentrations for 24, 48, and 72 h. Following each incubation period, 10 µL CellTiter-Blue Cell Viability Assay solution (Promega, Madison, WI, USA, Cat. No: G8080) was added to the wells, and the resazurin and resorufin absorbances were measured at a wavelength of 570 nm and 600 nm using a microplate reader. The IC_50_ value of temozolomide was calculated by using the absorbance data using GraphPad Prism V8 software.

### Experimental groups

The experimental design included the following groups: (1) an untreated control group, (2) a group exposed to temozolomide at its IC_50_ concentration, (3) a group transfected with anti-miR- 155 - 5p, (4) a negative control group transfected with a non-targeting anti-miR, (5) a group co-treated with temozolomide and anti-miR- 155 - 5p.

### Apoptosis assay

To assess the apoptotic consequences of miR- 155 - 5p silencing, all experimental groups described above were established. The Annexin V-FITC/PI Apoptosis Kit (Elabscience, Wuhan, China, Cat. No: E-CK-A211) and NovoCyte Flow Cytometer (Agilent, Santa Clara, CA, USA) were employed to determine apoptosis 24 h post-transfection. Following the manufacturer’s protocol. Data acquisition was performed using the PE and FITC channels, with a minimum of 20,000 events recorded per sample. Statistical comparisons among groups were performed via GraphPad Prism V8.

### Cell cycle assay

To evaluate the effects of miR- 155 - 5p silencing on the cell cycle, all previously described experimental groups were established. Cell cycle distribution was assessed 24 h post-transfection using the Cell Cycle Assay Kit (Elabscience, Cat. No: E-CK-A351) and analyzed with a NovoCyte Flow Cytometer (Agilent). Following the manufacturer’s instructions, cells were harvested, fixed, and stained with propidium iodide (PI) to allow DNA content analysis. Data acquisition was performed using the PE channel, with at least 20,000 events recorded per sample. Statistical comparisons between groups were conducted using GraphPad Prism V8.

### miRNA-mRNA Interaction, pathway, and survival analysis

The differentiated expression levels of miR- 155 - 5p in glioblastoma and normal brain tissues were analyzed using RPM (To compare gene expression levels across different samples regardless of sequencing depth, Reads Per Million (RPM) is calculated by dividing the raw read count of a given transcript by the total number of mapped reads in the sample and multiplying by 10^6^.) data from the miTED database, derived from the sequence read archive (SRA) data of 16 GBs and 31 normal brain tissue samples (Supplementary Table 3) (https://dianalab.e-ce.uth.gr/mited/#/; accessed in October 2024) (Kavakiotis et al. 2022). The target mRNAs of the miR- 155 - 5p were identified using the TargetScan Database (Version 8) (https://www.targetscan.org/vert_80/; accessed in October 2024) (Supplementary Table 4) (McGeary et al. 2019). The identified target genes (n = 571) were further analyzed through the STRING V12.0 database (https://string-db.org/; accessed in October 2024) to enrich GO Biological Processes, KEGG pathway, DISEASES terms (*p* < 0.05, interaction score = 0.7) (Szklarczyk et al. 2023). To assess the expression levels of mRNA targets of miR- 155 - 5p in glioblastoma, fold change of the expression levels between glioblastoma (n = 100) and normal (n = 10) tissues was obtained from the Human Protein Atlas database (Supplementary Table 5) (https://www.proteinatlas.org/; accessed in October 2024) (Sjöstedt et al. 2020). Visualizations of these networks were generated using Cytoscape 3.10.1 (Doncheva et al. 2018) and the STRING V12.0 database. Survival analyses were conducted to evaluate the prognostic significance of genes with decreased expression in glioblastoma (GBM) among the mRNAs targeted by miR- 155 - 5p. The analyses were performed using GEPIA (http://gepia.cancer-pku.cn/index.html; accessed in October 2024), which utilizes RNA sequencing data from 163 brain tumor tissues in TCGA database. The survival analysis was based on the Cox Proportional Hazards (Cox PH) Model, and hazard ratios (HRs) were calculated with a 95% confidence interval (CI) to assess the impact of gene expression on patient survival outcomes. A median-based group cutoff was applied to stratify patients into high- and low-expression groups. Overall survival (OS) was used as the primary endpoint (Tang et al. 2017).

### Statistical analyses

All experiments were conducted with a minimum of three biological replicates. Statistical analyses were performed using GraphPad Prism V8 software. For comparisons between two groups, an unpaired t-test was employed, while one-way ANOVA was used for comparisons involving more than two groups. A p-value of less than 0.05 was considered statistically significant.

## Results

### miR- 155 - 5p is significantly upregulated in glioblastoma tissues and brain cancer stem cells

**Fig. 1:**
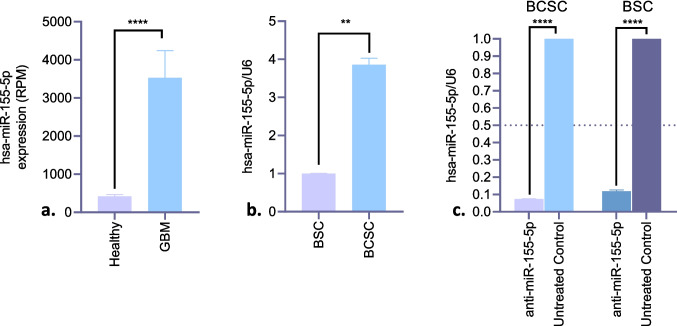
Expression levels of miR- 155 - 5p. **a**) Expression levels in glioblastoma and healthy brain tissues (To compare gene expression levels across different samples regardless of sequencing depth, Reads Per Million (RPM) is calculated by dividing the raw read count of a given transcript by the total number of mapped reads in the sample and multiplying by 10^6^.); **b**) Expression levels in BCSCs compared to BSCs (RPM: Reads per million); **c)** Expression levels in anti-miR- 155 - 5p treated BCSCs and BSCs compared to untreated control cells Statistical significance was evaluated by Student’s t-test (*p* < 0.05: *, *p* < 0.01: **, *p* < 0.001: ***, *p* < 0.0001: ****)

Analysis of the miTED database revealed a significant upregulation of miR- 155 - 5p in glioblastoma tissues compared to healthy brain tissues. The mean expression level of miR- 155 - 5p in glioblastoma samples (n = 16) was 3529, whereas in healthy brain tissues (n = 31), it was 415.9 (*p* < 0.0001; Fig. 1a). This result represents an 8.49-fold miR- 155 - 5p expression increase in glioblastoma. qPCR analysis showed that miR- 155 - 5p upregulated 3.858-fold in BCSCs compared to BSCs (*p* < 0.005; Fig. 1b). qPCR was performed for validation of RNA interference efficiency. In BCSCs and BSCs treated with anti-miR- 155 - 5p, miR- 155a- 5p expression levels were reduced by 13.42-fold and 8.50-fold, respectively, in comparison to the untreated control groups (*p* < 0.0001; Fig. 1c).

### Temozolomide exhibits cytotoxic effects on brain cancer stem cells exclusively at high concentrations

**Fig. 2:**
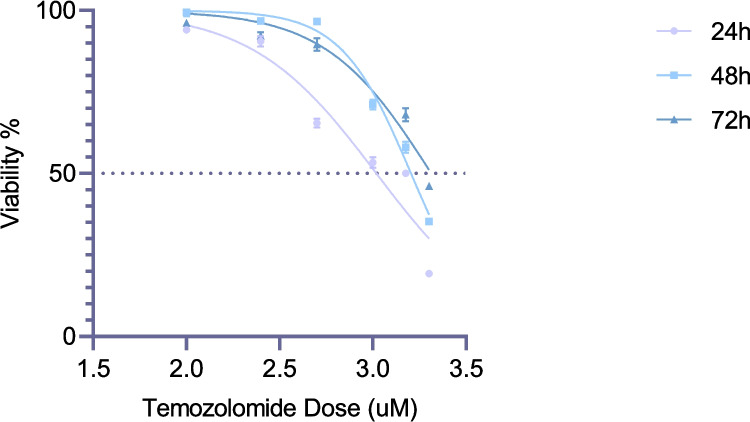
Effects of temozolomide on brain cancer stem cell viability at 24 th, 48 th, and 72nd hours

The cytotoxic effects of temozolomide on BCSCs were evaluated at 24, 48, and 72 h. IC_50_ values were determined as 1044 µM (R^2^ = 0.9168), 1601 µM (R^2^ = 0.984), and 2058 µM (R^2^ = 0.9477), respectively (Fig. 2). These results indicate a time-dependent increase in the IC_50_ values, suggesting that higher temozolomide concentrations are required to achieve similar cytotoxic effects over extended treatment durations. Based on these findings, subsequent experiments were conducted using a concentration of 1044 µM for 24 h.

### Anti-miR- 155 - 5p Enhances the apoptotic impact of temozolomide on brain cancer stem cells

**Fig. 3:**
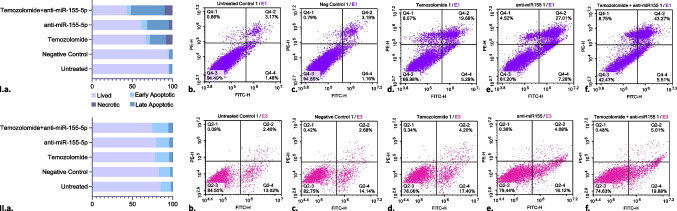
Annexin V assay results of the I) Brain Cancer Stem and II) Brain Stem Cells. **a**) Sum of the results. The different colors in the bar graphs represent distinct cell populations: light purple indicates live cells, light blue represents early apoptotic cells, dark blue corresponds to late apoptotic cells, and dark purple denotes necrotic cells. **b**) Untreated **c**) Negative Control Treated **d**) Temozolomide Treated **e**) anti-miR- 155 - 5p Treated **f**) anti-miR- 155 - 5p and Temozolomide Treated Cells

The Annexin V assay results demonstrated distinct differences in cell viability and apoptotic/necrotic profiles among the experimental groups. Figure 3I presents the results obtained from brain cancer stem cells (BCSCs), while Fig. 3II corresponds to brain stem cells (BSCs). In the untreated group of BSCSs, the percentage of live cells was 94.4%, with 4.7% apoptotic (early and late), and 0.8% necrotic cells (Fig. 3Ib). Similarly, in the negative control group, 94.2% of the cells were viable, with 5% apoptotic (early and late), and 0.8% necrotic cells (Fig. 3Ic). Statistical analysis revealed no significant differences (p > 0.999).

Temozolomide treatment resulted in a notable reduction in cell viability to 66.5%, representing a 1.42-fold decrease compared to the untreated cells (*p* < 0.0001). Early, late apoptotic, and necrotic cells increased 3.53, 6.25, and 10.25-fold, respectively (*p* < 0.0001; Fig. 3Id).

In the anti-miR- 155 - 5p treatment group, cell viability decreased to 61.2%, reflecting a 1.54-fold decrease compared to untreated cells (*p* < 0.0001). Early and late apoptotic cells increased 4.80-fold and 8.50-fold, respectively (*p* < 0.0001), and necrotic cells increased 5.38-fold (*p* = 0.0001; Fig. 3Ie).

The temozolomide and anti-miR- 155 - 5p combination further reduced cell viability to 42.9%, corresponding to a 2.20-fold decrease compared to untreated cells (*p* < 0.0001). Early apoptosis showed a 3.60-fold increase (*p* < 0.0001), while late apoptosis and necrosis increased 13.13-fold and 12.13-fold, respectively (*p* < 0.0001). The combination treatment represented a 1.55-fold decrease compared to temozolomide alone (*p* < 0.0001). Early apoptosis and necrosis remained relatively stable with a 1.02 and 1.18-fold increase (p > 0.999), whereas late apoptosis increased 2.10-fold (*p* < 0.0001), indicating a synergistic effect in promoting late apoptosis (Fig. 3If).

According to the Annexin V assay results, temozolomide and anti-miR- 155 - 5p treatments also led to different effects on healthy BSCs (Fig. 3IIa). In the untreated group of healthy control cells, 84.5% of the cells remained viable, with 13.02% in early apoptosis, 2.4% in late apoptosis, and 0.09% in necrosis. Similarly, the negative control group showed 82.75% viable cells, 14.14% early apoptotic, 2.68% late apoptotic, and 0.42% necrotic cells (p > 0.999; Fig. 3IIb).

Temozolomide caused a decrease in cell viability to 78.06%, reflecting a 1.08-fold reduction according to the untreated cells (*p* < 0.0001). Early apoptotic cells increased 1.34-fold (*p* = 0.026), late apoptotic cells increased 1.75-fold (*p* = 0.9677), and necrotic cells increased 3.78-fold (p > 0.9999) (Fig. 3IIc).

Anti-miR- 155 - 5p caused a 1.06-fold decrease in cell viability (*p* = 0.003). Early apoptosis increased 1.24-fold (*p* = 0.3767), late apoptosis 1.70-fold (*p* = 0.9834), and necrosis 4.00-fold (p > 0.9999) (Fig. 3IId).

The combination treatment further reduced cell viability to 74.63%, corresponding to a 1.13-fold decrease (*p* < 0.0001). Early, late apoptotic, and necrotic cells increased 1.53 (*p* < 0.0001), 2.09 (*p* = 0.6042), and 5.33 (*p* > 0.9999) folds, respectively (Fig. 3IIe).

When compared to the combination group to the temozolomide group, treatment resulted in a 1.05-fold decrease in cell viability (*p* = 0.1693), with early apoptosis increasing 1.14-fold (*p* = 0,6872), late apoptosis increasing 1.19-fold (p > 0.9999), and necrosis increasing 1.41-fold (p > 0.9999) (Fig. 3IIf). Overall, these findings indicate that anti-mir- 155 - 5p in combination with temozolomide did not cause statistically significant changes in healthy BSCs compared to the temozolomide treatment.

### Anti-miR- 155 - 5p and Temozolomide Combination Treatment Induces G2 Phase Arrest in Brain Cancer Stem Cells

**Fig. 4:**
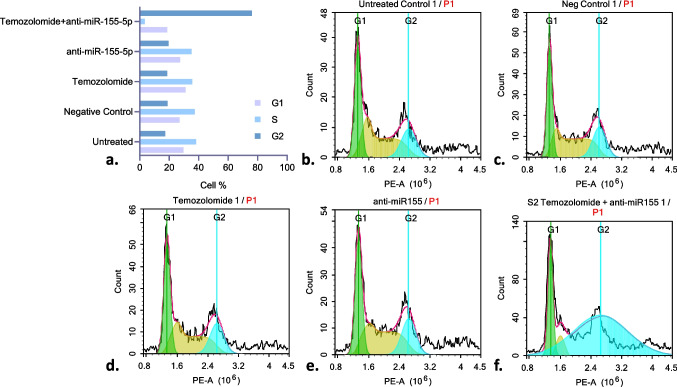
Cell cycle assay results of the Brain Cancer Stem Cells. **a**) Sum of the results. The G1 phase (light purple) represents the initial growth phase before DNA replication, the S phase (light blue) corresponds to DNA synthesis, and the G2 phase (dark blue) is the second growth phase before mitosis. **b**) Untreated **c**) Negative Control Treated **d**) Temozolomide Treated **e**) anti-miR- 155 - 5p Treated **f**) anti-miR- 155 - 5p and Temozolomide Treated Cells

The cell cycle analysis revealed significant differences among the experimental groups in the G1, S, and G2 phases distribution of cells (Fig. 4a). In the untreated group, the cell population was distributed as follows: 29.66% in the G1 phase, 38.12% in the S phase, and 17.28% in the G2 phase (Fig. 4b). The negative control group exhibited a similar distribution, with 27.04% in G1 (a 1.1-fold decrease compared to the untreated cells; *p* = 0.0039), 37.22% in S (a 1.02-fold decrease; *p* = 0.9417), and 18.89% in G2 (a 1.09-fold increase; *p* = 0.2563) (Fig. 4c).

Temozolomide treatment modestly increased the G1 phase to 31.07% (a 1.05-fold increase compared to the untreated group; *p* = 0.4496), while the S phase decreased to 35.64% (a 1.07-fold decrease; *p* = 0.0076). The G2 phase increased to 18.68% (a 1.08-fold increase; *p* = 0.4607). These changes suggest that temozolomide induces G1 phase arrest while reducing DNA synthesis (Fig. 4d).

Anti-miR- 155 - 5p treatment resulted in 29.05% of cells in G1 (a 1.02-fold decrease compared to the untreated cells; *p* = 0.9981), 36.08% in S phase (a 1.06-fold decrease; *p* = 0.0527), and 18.6% in G2 (a 1.08-fold increase; *p* = 0.5525), indicating that anti-miR- 155 - 5p has a minimal effect on cell cycle progression (Fig. 4e).

The combination of temozolomide and anti-miR- 155 - 5p induced a marked shift in cell cycle distribution, with a significant decrease in the G1 phase to 18.65% (a 1.67-fold decrease compared to temozolomide alone; *p* < 0.0001), a dramatic reduction in the S phase to 3.32% (a 10.73-fold decrease; *p* < 0.0001), and a substantial accumulation of cells in the G2 phase at 76.1% (a 4.07-fold increase; *p *< 0.0001). These results suggest a strong G2/M phase arrest induced by the combination treatment, indicating enhanced inhibition of cell cycle progression (Fig. 4f).

Overall, these findings indicate that temozolomide and anti-miR- 155 - 5p, particularly in combination, significantly alter cell cycle distribution. The combination treatment exerts the most pronounced effect by inducing G2 phase arrest and inhibiting DNA synthesis.

### miR- 155 - 5p might Control Various mRNAs Downregulated in Glioblastoma

Among the 10,764 genes with determined expression levels in glioblastoma, filtering for significantly downregulated genes (log2 fold change > 2.00, *p* < 0.05) identified 1,824 mRNAs. The genes exhibiting the most significant expression reduction were *GABRA1* (379.717), *SST* (384.304), *GABRD* (398.955), *TMEM240* (403.257), and *LY6H* (431.522) (Table 1).

**Table 1:** Fold changes and p values ​​of genes with the most decreased expression in glioblastoma (n = 50)

Gene	Gene name	p-value adjusted	logFC
ENSG00000176956	LY6H	0.002	− 431.52
ENSG00000205090	TMEM240	0.010	− 403.26
ENSG00000187730	GABRD	0.011	− 398.96
ENSG00000157005	SST	0.003	− 384.30
ENSG00000022355	GABRA1	0.002	− 379.72
ENSG00000127561	SYNGR3	0.002	− 378.82
ENSG00000100321	SYNGR1	0.002	− 377.60
ENSG00000145920	CPLX2	0.002	− 363.27
ENSG00000167371	PRRT2	0.002	− 361.42
ENSG00000183807	FAM162B	0.024	− 361.12
ENSG00000140600	SH3GL3	0.002	− 360.08
ENSG00000124507	PACSIN1	0.002	− 352.58
ENSG00000089169	RPH3 A	0.002	− 352.50
ENSG00000187902	SHISA7	0.002	− 352.33
ENSG00000176884	GRIN1	0.002	− 351.24
ENSG00000118733	OLFM3	0.003	− 348.65
ENSG00000160932	LY6E	0.002	− 347.33
ENSG00000124140	SLC12 A5	0.002	− 346.73
ENSG00000132702	HAPLN2	0.002	− 345.40
ENSG00000185518	SV2B	0.002	− 343.99
ENSG00000122733	PHF24	0.002	− 341.14
ENSG00000197283	SYNGAP1	0.002	− 340.85
ENSG00000113327	GABRG2	0.002	− 339.56
ENSG00000175497	DPP10	0.002	− 339.24
ENSG00000011083	SLC6 A7	0.002	− 338.93
ENSG00000198910	L1 CAM	0.002	− 337.81
ENSG00000168481	LGI3	0.002	− 337.21
ENSG00000164076	CAMKV	0.002	− 336.85
ENSG00000067715	SYT1	0.002	− 336.38
ENSG00000157445	CACNA2D3	0.002	− 336.31
ENSG00000136531	SCN2 A	0.002	− 336.26
ENSG00000149575	SCN2B	0.002	− 335.67
ENSG00000172260	NEGR1	0.002	− 334.44
ENSG00000180155	LYNX1	0.002	− 333.72
ENSG00000145864	GABRB2	0.002	− 333.19
ENSG00000151834	GABRA2	0.002	− 332.85
ENSG00000221946	FXYD7	0.002	− 332.06
ENSG00000142408	CACNG8	0.002	− 332.00
ENSG00000144285	SCN1 A	0.002	− 331.91
ENSG00000124785	NRN1	0.002	− 331.67
ENSG00000164326	CARTPT	0.017	− 331.48
ENSG00000114279	FGF12	0.002	− 331.43
ENSG00000102003	SYP	0.002	− 331.41
ENSG00000174469	CNTNAP2	0.002	− 331.17
ENSG00000164082	GRM2	0.002	− 331.10
ENSG00000163630	SYNPR	0.002	− 328.89
ENSG00000143858	SYT2	0.002	− 328.76
ENSG00000157782	CABP1	0.002	− 327.59
ENSG00000197430	OPALIN	0.005	− 326.64
ENSG00000121742	GJB6	0.004	− 324.73

**Fig. 5:**
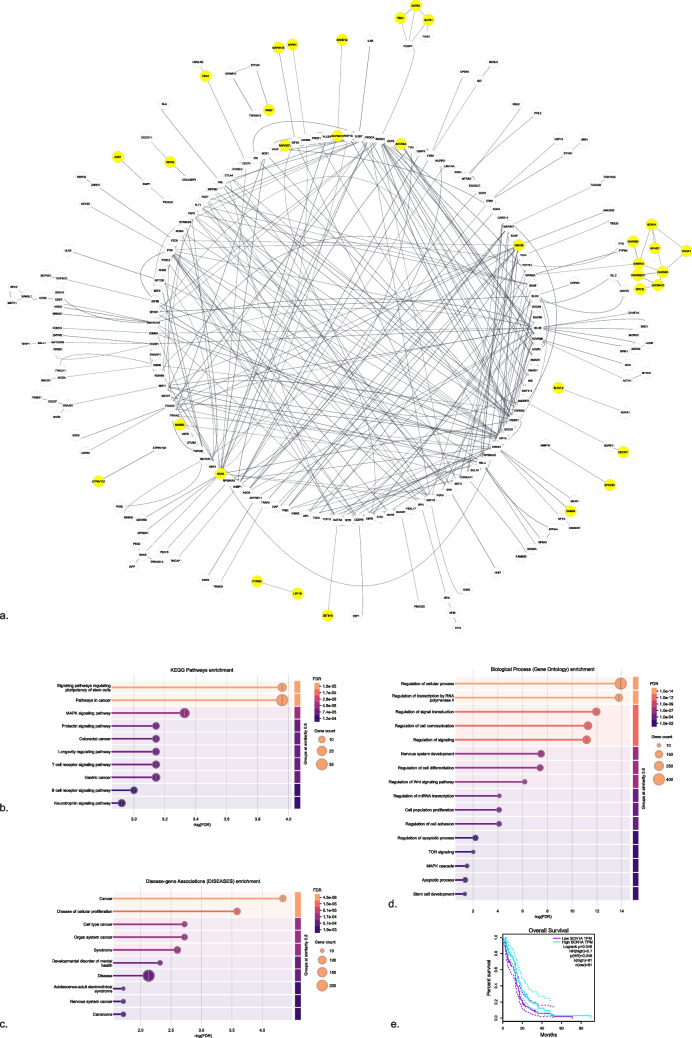
Target genes of miR- 155 - 5p; yellow bubbles indicate genes downregulated in glioblastoma (**a**). Terms regulated by target genes of miR- 155 - 5p; KEGG pathway (**b**), DISEASES (**c**), GO Biological Processes (**d**). The survival analysis of the SCN1 A gene, a target mRNA of miR- 155 - 5p, was conducted in glioblastoma. SCN1 A is downregulated in glioblastoma tissue samples compared to normal tissues (**e**)

Network analysis of the transcripts targeted by miR- 155 - 5p showed interactions (Fig. 5a; enrichment p-value < 1.0e- 16). The most enriched KEGG pathway terms included"Signaling pathways regulating pluripotency of stem cells"and"Pathways in cancer"(Fig. 5b). Among the DISEASES term enrichments,"Cancer"and"Nervous system cancer"were identified as highly significant (Fig. 5c). Additionally, GO Biological Processes analysis revealed enrichments not only in RNA and transcription regulation but also in terms related to"Nervous system development,""Cell population proliferation,""Regulation of apoptotic process,"and"Stem cell development"(Fig. 5d).

By identifying the intersection of transcripts targeted by miR- 155 - 5p and significantly downregulated mRNAs in glioblastoma (log2 fold change > 2.00, *p* < 0.05), 67 common genes were found. The most significantly downregulated genes in this group included *GABRA1* (379.72), *GABRB2* (333.199), *SCN1 A* (331.91), *GRIN2 A* (316.16), and *SGIP1* (313.73) (Table 2, yellow bubbles in Fig. 5a).

**Table 2:** Common genes that show decreased expression in glioblastoma and are targeted by miR- 155 - 5p (n = 67)

Gene	Target gene	logFC	p-value adjusted	3P-seq tags + 5	Conserved sites total	Conserved 8 mer sites	Conserved 7 mer-m8 sites	Conserved 7 mer-A1 sites	Poorly conserved sites total	Poorly conserved 8 mer sites	Poorly conserved 7 mer-m8 sites	Poorly conserved 7 mer-A1 sites	6 mer sites	Cumulative weighted context + + score	Total context + + score	Aggregate PCT
ENSG00000002746	HECW1	− 22.16	0.00	11	1	1	0	0	0	0	0	0	0	− 0.01	− 0.03	0.19
ENSG00000022355	GABRA1	− 379.72	0.00	5	1	1	0	0	2	0	2	0	1	− 0.46	− 0.46	0.37
ENSG00000046653	GPM6B	− 182.31	0.00	135	1	1	0	0	0	0	0	0	1	− 0.03	− 0.28	0.38
ENSG00000050426	LETMD1	− 154.33	0.00	1619	1	0	1	0	0	0	0	0	0	0	− 0.17	0.23
ENSG00000066248	NGEF	− 228.95	0.00	13	1	0	0	1	0	0	0	0	0	− 0.24	− 0.24	0.58
ENSG00000072518	MARK2	− 109.66	0.00	845	1	0	0	1	0	0	0	0	0	− 0.05	− 0.05	0.36
ENSG00000076554	TPD52	− 173.52	0.00	4585	1	0	0	1	0	0	0	0	0	− 0.19	− 0.19	0.41
ENSG00000082701	GSK3B	− 123.71	0.00	498	1	0	0	1	0	0	0	0	0	− 0.03	− 0.11	0.49
ENSG00000091428	RAPGEF4	− 268.36	0.00	60	1	0	0	1	0	0	0	0	0	− 0.13	− 0.13	0.67
ENSG00000091622	PITPNM3	− 223.88	0.00	5	1	0	1	0	0	0	0	0	0	− 0.13	− 0.13	0.23
ENSG00000102181	CD99L2	− 179.86	0.00	303	1	0	0	1	0	0	0	0	0	− 0.12	− 0.12	0.58
ENSG00000107105	ELAVL2	− 190.14	0.00	78	1	0	0	1	0	0	0	0	0	− 0.06	− 0.07	0.41
ENSG00000107560	RAB11 FIP2	− 169.22	0.00	449	2	1	1	0	0	0	0	0	1	− 0.39	− 0.45	0.49
ENSG00000107957	SH3PXD2 A	− 152.19	0.00	152	1	0	0	1	0	0	0	0	3	− 0.05	− 0.06	0.54
ENSG00000109756	RAPGEF2	− 17.16	0.00	484	1	1	0	0	0	0	0	0	2	− 0.22	− 0.22	0.15
ENSG00000109906	ZBTB16	− 11.02	0.00	73	1	1	0	0	0	0	0	0	0	− 0.17	− 0.18	0.52
ENSG00000111262	KCNA1	− 299.34	0.00	5	1	0	1	0	1	0	0	1	0	− 0.08	− 0.08	0.3
ENSG00000115042	FAHD2 A	− 115.20	0.00	9	1	1	0	0	0	0	0	0	0	− 0.12	− 0.22	0.84
ENSG00000115977	AAK1	− 184.71	0.00	27	1	0	1	0	2	0	2	0	3	− 0.15	− 0.2	0.23
ENSG00000118473	SGIP1	− 313.73	0.00	180	1	0	1	0	1	1	0	0	1	− 0.2	− 0.39	0.23
ENSG00000119042	SATB2	− 104.10	0.03	820	1	1	0	0	1	0	1	0	0	− 0.1	− 0.12	0.23
ENSG00000119401	TRIM32	− 122.47	0.00	285	1	1	0	0	0	0	0	0	0	− 0.39	− 0.4	0.29
ENSG00000119729	RHOQ	− 108.08	0.01	179	1	0	0	1	0	0	0	0	0	− 0.09	− 0.09	0.5
ENSG00000121989	ACVR2 A	− 158.66	0.18	109	1	0	1	0	0	0	0	0	0	− 0.05	− 0.12	0.23
ENSG00000127540	UQCR11	− 225.22	0.00	34490	1	0	0	1	0	0	0	0	0	− 0.24	− 0.24	0.5
ENSG00000130758	MAP3 K10	− 16.58	0.00	178	1	1	0	0	0	0	0	0	0	− 0.39	− 0.39	0.26
ENSG00000131437	KIF3 A	− 109.21	0.00	33	1	0	1	0	1	0	1	0	0	− 0.16	− 0.22	0.41
ENSG00000133275	CSNK1G2	− 109.98	0.00	5	1	1	0	0	0	0	0	0	0	− 0.31	− 0.31	0.66
ENSG00000133703	KRAS	− 133.80	0.00	40	1	0	1	0	1	0	0	1	0	− 0.26	− 0.31	0.27
ENSG00000134709	HOOK1	− 223.92	0.00	804	1	0	1	0	0	0	0	0	0	− 0.1	− 0.11	0.23
ENSG00000136535	TBR1	− 19.96	0.00	5	1	1	0	0	1	1	0	0	0	− 0.28	− 0.28	< 0.1
ENSG00000138411	HECW2	− 201.23	0.00	28	1	1	0	0	0	0	0	0	1	− 0.02	− 0.05	< 0.1
ENSG00000140199	SLC12 A6	− 138.95	0.00	17	1	0	1	0	2	1	1	0	0	− 0.19	− 0.22	0.23
ENSG00000144285	SCN1 A	− 331.91	0.00	21	1	0	1	0	0	0	0	0	0	− 0.11	− 0.11	0.23
ENSG00000144619	CNTN4	− 209.03	0.00	191	1	0	1	0	0	0	0	0	1	− 0.17	− 0.18	0.23
ENSG00000145087	STXBP5L	− 303.14	0.00	19	1	1	0	0	0	0	0	0	0	− 0.15	− 0.15	< 0.1
ENSG00000145730	PAM	− 128.61	0.00	17,149	1	0	1	0	0	0	0	0	1	0	− 0.02	0.23
ENSG00000145864	GABRB2	− 333.19	0.00	5	1	0	0	1	1	0	1	0	0	− 0.18	− 0.18	0.52
ENSG00000151067	CACNA1 C	− 143.57	0.00	69	1	0	0	1	0	0	0	0	0	− 0.04	− 0.06	0.72
ENSG00000152092	ASTN1	− 167.96	0.00	9	1	0	1	0	0	0	0	0	0	− 0.1	− 0.1	0.23
ENSG00000152377	SPOCK1	− 210.80	0.00	935	1	1	0	0	0	0	0	0	0	− 0.19	− 0.2	< 0.1
ENSG00000152495	CAMK4	− 279.93	0.00	73	1	1	0	0	0	0	0	0	4	− 0.17	− 0.19	0.12
ENSG00000152642	GPD1L	− 139.57	0.00	130	1	1	0	0	0	0	0	0	0	− 0.1	− 0.12	0.15
ENSG00000153707	PTPRD	− 165.50	0.00	101	1	0	1	0	0	0	0	0	0	− 0.08	− 0.15	0.23
ENSG00000153956	CACNA2D1	− 259.20	0.00	418	1	0	1	0	1	0	0	1	0	− 0.03	− 0.03	0.25
ENSG00000155097	ATP6 V1 C1	− 187.56	0.00	951	1	0	0	1	0	0	0	0	1	− 0.18	− 0.2	0.47
ENSG00000155974	GRIP1	− 201.72	0.00	28	1	0	0	1	0	0	0	0	1	− 0.11	− 0.19	0.34
ENSG00000158560	DYNC1I1	− 186.80	0.00	25	1	1	0	0	0	0	0	0	0	− 0.48	− 0.48	0.4
ENSG00000162105	SHANK2	− 261.59	0.00	26	1	0	1	0	1	0	1	0	1	− 0.05	− 0.09	0.23
ENSG00000162374	ELAVL4	− 165.98	0.00	5	1	0	1	0	0	0	0	0	0	− 0.07	− 0.07	0.23
ENSG00000163531	NFASC	− 266.32	0.00	10	1	0	0	1	0	0	0	0	0	− 0.06	− 0.06	0.64
ENSG00000165898	ISCA2	− 117.83	0.00	135	1	0	1	0	2	0	1	1	0	− 0.02	− 0.52	0.23
ENSG00000168702	LRP1B	− 108.49	0.01	26	2	1	1	0	0	0	0	0	0	− 0.11	− 0.13	0.38
ENSG00000169213	RAB3B	− 229.20	0.00	611	1	0	1	0	3	0	1	2	0	− 0.05	− 0.26	0.34
ENSG00000169891	REPS2	− 194.83	0.00	36	1	0	1	0	0	0	0	0	0	− 0.02	− 0.16	0.23
ENSG00000170989	S1PR1	− 111.76	0.13	251	1	0	1	0	1	1	0	0	0	− 0.25	− 0.66	0.23
ENSG00000171033	PKIA	− 220.84	0.00	542	1	0	1	0	2	0	0	2	0	− 0.25	− 0.31	0.24
ENSG00000171951	SCG2	− 162.21	0.01	55	1	0	1	0	0	0	0	0	0	− 0.33	− 0.33	0.23
ENSG00000176658	MYO1D	− 147.60	0.04	16	1	1	0	0	0	0	0	0	0	− 0.36	− 0.36	0.56
ENSG00000177570	SAMD12	− 179.71	0.18	57	1	0	1	0	1	0	1	0	0	− 0.3	− 0.3	0.41
ENSG00000182389	CACNB4	− 2.74	0.00	57	1	0	1	0	1	0	0	1	2	− 0.05	− 0.14	0.42
ENSG00000182568	SATB1	− 110.50	0.01	312	1	0	1	0	1	0	1	0	0	− 0.22	− 0.24	0.23
ENSG00000183454	GRIN2 A	− 316.16	0.00	5	1	1	0	0	2	0	2	0	3	0	− 0.07	0.3
ENSG00000196776	CD47	− 295.33	0.00	520	1	0	1	0	1	0	0	1	1	− 0.11	− 0.29	0.25
ENSG00000196850	PPTC7	− 145.53	0.00	817	1	0	0	1	0	0	0	0	0	− 0.13	− 0.13	0.34
ENSG00000197147	LRRC8B	− 20.03	0.00	326	1	1	0	0	1	0	1	0	1	− 0.11	− 0.23	< 0.1
ENSG00000206579	XKR4	− 225.89	0.00	5	1	0	0	1	3	0	1	2	0	− 0.33	− 0.33	0.72

Survival analysis was conducted for all common genes to evaluate their prognostic significance in glioblastoma (Table 3). Notably, low expression of *SCN1 A* was found to be associated with poor prognosis (*p* < 0.05; HR = 0.7), suggesting its potential role as a prognostic marker (Fig. 5e).

**Table 3:** Survival analysis results

Gene	Survival analyses results
AAK1	Logrank *p *= 0.95 HR(high) = 1 p(HR) = 0.96 n(high) = 81 n(low) = 81
ACVR2 A	Logrank *p* = 0.94 HR(high) = 0.99 p(HR) = 0.96 n(high) = 81 n(low) = 81
ASTN1	Logrank *p* = 0.9 HR(high) = 1 p(HR) = 0.9 n(high) = 81 n(low) = 81
ATP6 V1 C1	Logrank *p* = 0.58 HR(high) = 1.1 p(HR) = 0.57 n(high) = 81 n(low) = 81
CACNA1 C	Logrank *p* = 0.36 HR(high) = 1.2 p(HR) = 0.36 n(high) = 80 n(low) = 80
CACNA2D1	Logrank *p* = 0.76 HR(high) = 1.1 p(HR) = 0.78 n(high) = 81 n(low) = 81
CACNB4	Logrank *p* = 0.78 HR(high) = 0.95 p(HR) = 0.78 n(high) = 80 n(low) = 81
CAMK4	Logrank *p* = 0.022 HR(high) = 1.5 p(HR) = 0.023 n(high) = 79 n(low) = 81
CD47	Logrank *p* = 0.22 HR(high) = 1.2 p(HR) = 0.22 n(high) = 81 n(low) = 81
CD99L2	Logrank *p* = 0.54 HR(high) = 1.1 p(HR) = 0.54 n(high) = 81 n(low) = 81
CNTN4	Logrank *p* = 0.38 HR(high) = 1.2 p(HR) = 0.37 n(high) = 80 n(low) = 80
CSNK1G2	Logrank *p *= 1 HR(high) = 1 p(HR) = 0.98 n(high) = 81 n(low) = 81
DYNC1I1	Logrank *p* = 0.9 HR(high) = 0.97 p(HR) = 0.88 n(high) = 80 n(low) = 81
ELAVL2	Logrank *p* = 0.85 HR(high) = 1 p(HR) = 0.87 n(high) = 81 n(low) = 81
ELAVL4	Logrank *p* = 0.65 HR(high) = 1.1 p(HR) = 0.67 n(high) = 81 n(low) = 81
FAHD2 A	Logrank *p* = 0.0054 HR(high) = 1.6 p(HR) = 0.0062 n(high) = 81 n(low) = 81
GABRA1	Logrank *p* = 0.15 HR(high) = 1.3 p(HR) = 0.16 n(high) = 81 n(low) = 81
GABRB2	Logrank *p* = 0.35 HR(high) = 1.2 p(HR) = 0.36 n(high) = 81 n(low) = 80
GPD1L	Logrank *p* = 0.15 HR(high) = 0.77 p(HR) = 0.15 n(high) = 81 n(low) = 80
GPM6B	Logrank *p* = 0.23 HR(high) = 0.81 p(HR) = 0.23 n(high) = 81 n(low) = 81
GRIN2 A	Logrank *p* = 0.32 HR(high) = 1.2 p(HR) = 0.33 n(high) = 81 n(low) = 81
GRIP1	Logrank *p* = 0.44 HR(high) = 1.1 p(HR) = 0.46 n(high) = 81 n(low) = 78
GSK3B	Logrank *p* = 0.16 HR(high) = 0.77 p(HR) = 0.16 n(high) = 81 n(low) = 81
HECW1	Logrank *p* = 0.19 HR(high) = 1.3 p(HR) = 0.2 n(high) = 81 n(low) = 81
HECW2	Logrank *p* = 0.96 HR(high) = 0.99 p(HR) = 0.94 n(high) = 81 n(low) = 81
HOOK1	Logrank *p* = 0.41 HR(high) = 0.86 p(HR) = 0.41 n(high) = 78 n(low) = 81
ISCA2	Logrank *p* = 0.43 HR(high) = 0.87 p(HR) = 0.45 n(high) = 81 n(low) = 81
KCNA1	Logrank *p* = 0.66 HR(high) = 1.1 p(HR) = 0.65 n(high) = 80 n(low) = 81
KIF3 A	Logrank *p* = 0.33 HR(high) = 0.84 p(HR) = 0.32 n(high) = 80 n(low) = 81
KRAS	Logrank *p* = 0.56 HR(high) = 0.9 p(HR) = 0.57 n(high) = 81 n(low) = 81
LETMD1	Logrank *p* = 0.34 HR(high) = 1.2 p(HR) = 0.37 n(high) = 81 n(low) = 81
LRP1B	Logrank *p* = 0.47 HR(high) = 0.88 p(HR) = 0.46 n(high) = 81 n(low) = 81
LRRC8B	Logrank p = 0.5 HR(high) = 0.89 p(HR) = 0.51 n(high) = 81 n(low) = 81
MAP3 K10	Logrank *p* = 0.46 HR(high) = 1.1 p(HR) = 0.47 n(high) = 81 n(low) = 81
MARK2	Logrank *p* = 0.96 HR(high) = 1 p(HR) = 0.95 n(high) = 81 n(low) = 81
MYO1D	Logrank *p* = 0.22 HR(high) = 1.2 p(HR) = 0.23 n(high) = 81 n(low) = 81
NFASC	Logrank *p* = 0.43 HR(high) = 1.1 p(HR) = 0.45 n(high) = 81 n(low) = 81
NGEF	Logrank *p* = 0.088 HR(high) = 1.4 p(HR) = 0.087 n(high) = 81 n(low) = 81
PAM	Logrank *p* = 0.19 HR(high) = 1.3 p(HR) = 0.18 n(high) = 81 n(low) = 81
PITPNM3	Logrank *p* = 0.58 HR(high) = 1.1 p(HR) = 0.57 n(high) = 81 n(low) = 81
PKIA	Logrank *p* = 0.45 HR(high) = 1.1 p(HR) = 0.48 n(high) = 81 n(low) = 81
PPTC7	Logrank *p* = 0.63 HR(high) = 0.92 p(HR) = 0.64 n(high) = 81 n(low) = 81
PTPRD	Logrank *p* = 0.81 HR(high) = 0.96 p(HR) = 0.81 n(high) = 81 n(low) = 81
RAB11 FIP2	Logrank *p* = 0.59 HR(high) = 0.91 p(HR) = 0.6 n(high) = 81 n(low) = 80
RAB3B	Logrank *p* = 0.48 HR(high) = 1.1 p(HR) = 0.47 n(high) = 81 n(low) = 79
RAPGEF2	Logrank *p* = 0.32 HR(high) = 0.83 p(HR) = 0.31 n(high) = 81 n(low) = 81
RAPGEF4	Logrank *p* = 0.98 HR(high) = 1 p(HR) = 0.98 n(high) = 81 n(low) = 79
REPS2	Logrank *p* = 0.31 HR(high) = 0.83 p(HR) = 0.32 n(high) = 81 n(low) = 81
RHOQ	Logrank *p* = 0.28 HR(high) = 0.82 p(HR) = 0.28 n(high) = 81 n(low) = 81
S1PR1	Logrank *p* = 0.62 HR(high) = 0.91 p(HR) = 0.59 n(high) = 81 n(low) = 81
SAMD12	Logrank *p* = 0.88 HR(high) = 1 p(HR) = 0.9 n(high) = 81 n(low) = 80
SATB1	Logrank *p* = 0.13 HR(high) = 1.3 p(HR) = 0.14 n(high) = 81 n(low) = 81
SATB2	Logrank *p* = 1 HR(high) = 1 p(HR) = 0.99 n(high) = 81 n(low) = 81
SCG2	Logrank *p* = 0.2 HR(high) = 1.3 p(HR) = 0.19 n(high) = 81 n(low) = 81
***SCN1A***	***Logrank p= 0.046 HR(high) = 0.7 p(HR) = 0.046 n(high) = 81 n(low) = 81***
SGIP1	Logrank *p* = 0.69 HR(high) = 1.1 p(HR) = 0.72 n(high) = 81 n(low) = 81
SH3PXD2 A	Logrank *p* = 0.85 HR(high) = 0.97 p(HR) = 0.85 n(high) = 81 n(low) = 81
SHANK2	Logrank *p* = 0.28 HR(high) = 1.2 p(HR) = 0.29 n(high) = 81 n(low) = 81
SLC12 A6	Logrank *p* = 0.86 HR(high) = 1 p(HR) = 0.86 n(high) = 80 n(low) = 81
SPOCK1	Logrank *p* = 0.29 HR(high) = 1.2 p(HR) = 0.29 n(high) = 81 n(low) = 81
STXBP5L	Logrank *p* = 0.74 HR(high) = 0.94 p(HR) = 0.72 n(high) = 81 n(low) = 81
TBR1	Logrank *p* = 0.11 HR(high) = 1.3 p(HR) = 0.12 n(high) = 81 n(low) = 78
TPD52	Logrank *p* = 0.37 HR(high) = 0.85 p(HR) = 0.37 n(high) = 81 n(low) = 81
TRIM32	Logrank *p* = 0.32 HR(high) = 0.84 p(HR) = 0.33 n(high) = 81 n(low) = 81
UQCR11	Logrank *p* = 0.25 HR(high) = 0.81 p(HR) = 0.25 n(high) = 81 n(low) = 81
XKR4	Logrank *p* = 0.82 HR(high) = 1 p(HR) = 0.85 n(high) = 81 n(low) = 80
ZBTB16	Logrank *p* = 0.98 HR(high) = 1 p(HR) = 0.98 n(high) = 81 n(low) = 81

**Fig. 6:**
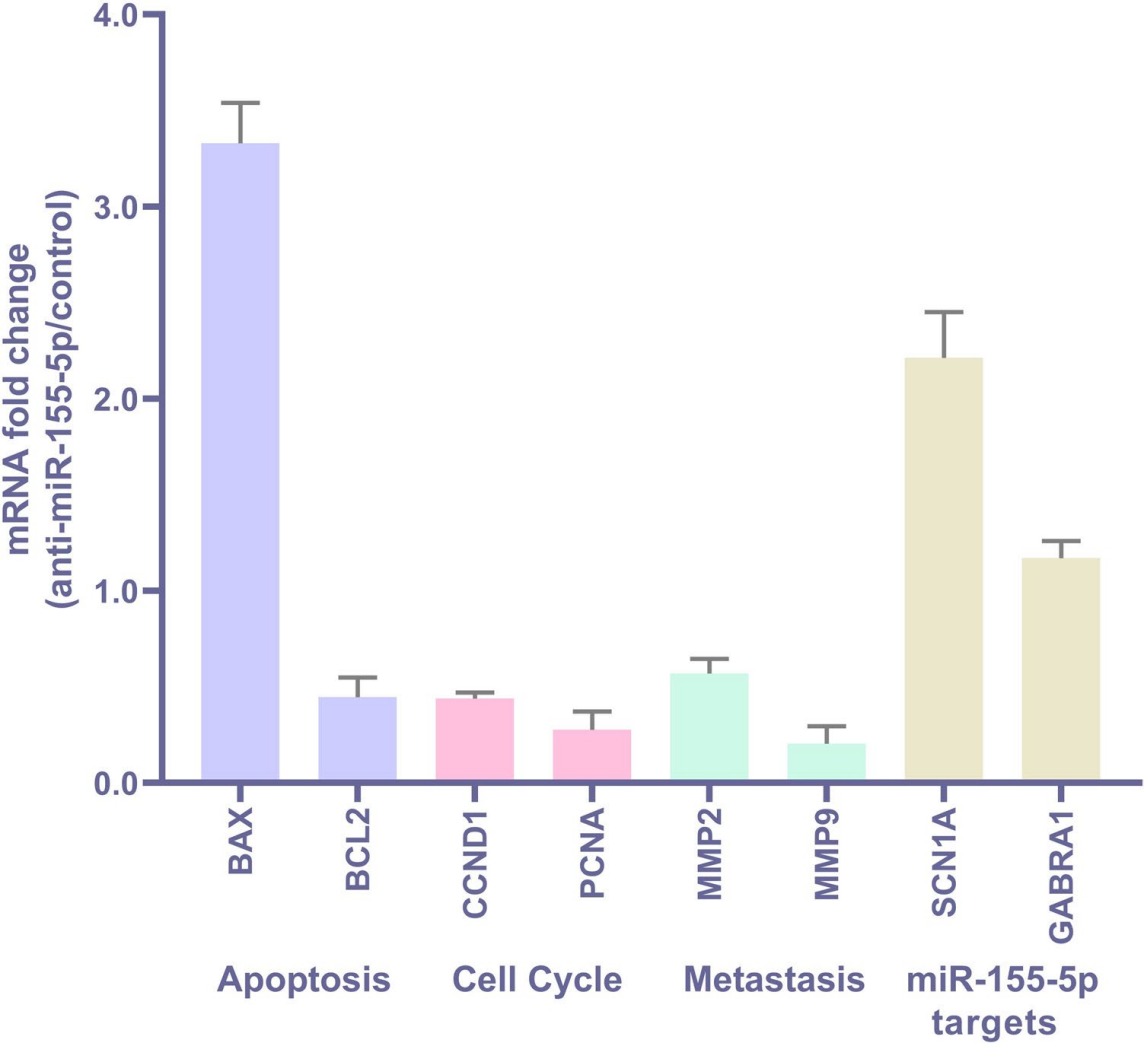
qPCR analysis of gene expression changes in brain cancer stem cells (BCSCs) following miR- 155 - 5p silencing. Relative mRNA expression levels of apoptosis-related genes (BAX, BCL2), cell cycle-related genes (CCND1, PCNA), metastasis-related genes (MMP2, MMP9) and potential downstream targets of miR- 155 - 5p (GABRA1, SCN1 A) were analyzed in BCSCs treated with anti-miR- 155 - 5p compared to negative control-treated cells. Statistical significance was evaluated by Student’s t-test (*p* < 0.05: *, *p* < 0.01: **, *p* < 0.001: ***, *p* < 0.0001: ****)

To gain preliminary insight into the molecular mechanisms underlying the phenotypic effects of miR- 155 - 5p silencing, the expression levels of selected genes involved in apoptosis, cell cycle regulation, and metastasis were assessed in BCSCs using qPCR (Fig. 6;Table 4). Among apoptosis-related genes, *BAX* expression was found to be 3.33-fold upregulated, while *BCL2* expression was 0.45-fold downregulated in anti-miR- 155 - 5p–treated BCSCs compared to negative controls, suggesting an apoptotic shift at the transcriptional level. In terms of cell cycle regulation, *CCND1* and *PCNA* expression levels were downregulated by 0.44-fold and 0.28-fold, respectively, indicating that miR- 155 - 5p inhibition may affect cell cycle progression. For metastasis-related markers, *MMP2* and *MMP9* expression showed a change of 0.57-fold and 0.20-fold, respectively, pointing to a potential reduction in invasive properties.

**Table 4:** qPCR analysis results

Gene	Fold change (anti-miR- 155 - 5p/negative control)	SD
BAX	3.33	0.21
BCL2	0.45	0.10
CCND1	0.44	0.03
PCNA	0.28	0.10
MMP2	0.57	0.08
MMP9	0.20	0.09
SCN1A	2.21	0.24
GABRA1	1.17	0.09

Additionally, the expression levels of *GABRA1* and *SCN1A*, two potential downstream targets of miR- 155 - 5p, were analyzed. *GABRA1* was 1.17-fold upregulated and *SCN1A* was 2.21-fold upregulated, indicating that these genes may be post-transcriptionally regulated by miR- 155 - 5p in BCSCs. These findings provide preliminary molecular evidence that miR- 155 - 5p silencing modulates key gene expression programs related to apoptosis, cell proliferation, and metastasis.

## Discussion

Glioblastoma is a progressive and aggressive cancer of the central nervous system that accounts for nearly 49% of brain-related carcinomas in adults (Schaff and Mellinghoff 2023). The reported median survival time from the date of diagnosis is shorter than one year. Despite chemotherapy with antitumoral temozolomide in glioblastoma patients, many patients experience TMZ resistance and aggressive tumor recurrence. The latest research focuses on the molecular mechanisms behind TMZ resistance, which inhibits the drug's cytotoxic effect, to identify new molecular targets to improve the prognosis of gliomas (Jezierzański et al. 2024).

MiR- 155 is a multifunctional miRNA that plays a role in physiological processes (proliferation, cell cycle, apoptosis, and differentiation), attracts attention with its overexpression in malignant tumors, and triggers mechanisms of treatment resistance in many tumor types (Bayraktar et al. 2018; Chen et al. 2020). Accumulating evidence has shown that MiR- 155 - 5p plays various roles in different types of cancer. MiR- 155 - 5p acts as an oncogene in non-small cell lung and breast cancers, colorectal, hepatocellular, and oral squamous cell carcinoma, and as a tumor suppressor in Wilms tumor. (Pagotto et al. 2018; Xue et al. 2016; Fu et al. 2017; Zhang et al. 2013; Rather et al. 2013; Jiang et al. 2010; Luo et al. 2020). miR- 155 - 5p regulated TP53INP1 expression in cervical cancer cells and was expressed at significantly high levels in cancer tissues, while downregulated miR- 155 - 5p expression suppressed tumor growth and metastasis (Li et al. 2019). Another study showed that miR- 155 - 5p enhances renal cancer metastasis and epithelial-mesenchymal transition by targeting apoptosis-inducing factors. In the same study, suppression of MiR- 155 - 5p decreased tumor growth, cell migration, and metastasis, while increasing E-cadherin levels (Lei et al. 2020). Although there are studies on various cancer types evaluating the potential of miR- 155 - 5p-based therapeutic approaches in the literature, studies investigating the role of TM found that the efficacy of TMZ used in glioblastoma chemotherapy is very limited. Accordingly, in this study, we found that miR- 155 - 5p was upregulated in brain cancer stem cells compared to healthy brain stem cells (3.58-fold, *p* < 0.0001). miTED database analysis revealed that miR- 155 - 5p was significantly overexpressed in glioblastoma tissues compared to healthy brain tissues (8.49-fold, *p* < 0.0001). These results are consistent with studies reporting that miR- 155 - 5p has an oncogenic function.

Temozolomide is a potent apoptosis inducer, inhibits tumor growth in a three-dimensional cell culture model, and develops resistance to treatment with low-dose applications in glioblastoma chemotherapy (Strobel et al. 2019). The adaptive properties and heterogeneity of glioblastoma cells are important for the efficacy of temozolomide treatment. This is because these features contribute to the tumor's capacity to endure, leading to the potentiation of apoptosis escape mechanisms and the appearance of subpopulations with acquired resistance mechanisms (Fabro et al. 2023). In the present study, the cytotoxic activity of temozolomide showed a time-dependent increase in IC_50_ values. This suggests that higher doses of temozolomide are required to achieve similar cytotoxic effects over longer treatment periods. The sensitivity of glioblastoma cells to TMZ varies depending on the cell line and experimental conditions. A systematic review reported that in the U87 glioblastoma cell line, the median IC_50_ values increased from 123.9 µM at 24 h to 223.1 µM at 48 h and 230.0 µM at 72 h, suggesting a time-dependent decrease in TMZ sensitivity (Poon et al. 2021). However, specific data on the time-dependent IC_50_ values in brain cancer stem cells (BCSCs) remain limited. Previous studies on glioma stem cells indicate that their inherent resistance to TMZ may be attributed to mechanisms such as enhanced DNA repair capacity and the upregulation of drug efflux transporters (Tomar et al. 2021). These adaptations may contribute to the observed reduction in TMZ efficacy over time. Further investigations are required to fully elucidate the temporal dynamics of TMZ-induced cytotoxicity in BCSCs and to determine the molecular mechanisms responsible for any changes in drug sensitivity.

In a recent study investigating the impact of the combination of hsa-miR- 34a- 5p and temozolomide on glioblastoma, hsa-miR- 34a- 5p was shown to potentiate the therapeutic efficacy of temozolomide by suppressing RAF1 in the MAPK pathway, inhibiting cell viability, migration and proliferation and increasing apoptosis in glioblastoma multiforme (Shadbad et al. 2024). In a study investigating the role of miR- 155 in treatment resistance in lung cancer, it was determined that anti-miR- 155 therapy may be a promising target in treatment-resistant cancers by resensitizing tumors to chemotherapy (Roosbroeck et al. 2017). Another study investigating the effects of combined treatment of two peptide nucleic acids (PNAs) directed against miR- 155 - 5p and miR- 221 - 3p in the TMZ-resistant T98G glioma cell line, co-administration of both anti-miR- 155 and anti-miR- 221 PNAs was associated with an increased proapoptotic activity (Milani et al. 2019). Similarly, in our study, cell viability was significantly reduced by 42.9% in the combined treatment (anti-miR- 155 - 5p + temozolomide) group compared to the untreated group. Early, and late apoptosis and necrosis were increased considerably (− 3.60, − 13.13, − 12.13, respectively). While TMZ treatment in combination with anti-miR- 155 - 5p showed a significant decrease in cell viability compared to TMZ alone, early apoptosis and necrosis remained relatively constant with 1.02-fold and 1.18-fold increases. In contrast, late apoptosis increased 2.10-fold suggesting a synergistic effect in promoting late apoptosis. In addition, it may be proposed that the combination of anti-miR- 155 - 5p and temozolomide provides a more potent anti-tumoral effect than the effect of temozolomide alone and enhances cell death, especially through late apoptosis. Our results also show that combining temozolomide and anti-miR- 155 - 5p inhibits DNA synthesis by arresting the cell cycle in the G2 phase. This effect may stop the proliferation of tumor cells by preventing cells from dividing, providing a strong anti-tumoral effect of the treatment.

In conclusion, the decrease in temozolomide's cytotoxic effect over time may be due not only to the drug's biological properties but also to cancer cells'dynamic adaptability. At the same time, this study identified that miR- 155 - 5p may play an important role in resistance to temozolomide chemotherapy against glioblastoma. Therefore, combining anti-miR- 155 - 5p with routine temozolomide treatment would be associated with a potent antitumoral effect and possibly fewer side effects, despite using smaller doses. So, miR- 155 - 5p may be a novel therapeutic target that can be downregulated in the conventional chemotherapy approach for brain cancer treatment. Consequently, to achieve long-term success in aggressive and progressive brain tumors such as glioblastoma, new therapeutic strategies targeting cancer stem cells in combination with temozolomide need to be developed.

Although our findings provide valuable insights into the role of anti-miR- 155 - 5p in temozolomide-resistant glioblastoma cells, there are some limitations. They were performed using BCSC and BCS cell lines, which, while informative, may not fully capture the heterogeneity of glioblastoma. Furthermore, to assess the therapeutic potential of anti-miR- 155 - 5p, in vivo validation with further preclinical studies, including animal models, is required. Another limitation of the study, specific downstream targets or pathways that may be affected due to deletion of miR- 155 - 5p were not investigated in detail. These will be essential to evaluate the efficacy and safety of this strategy before moving into clinical practice.

## Supplementary Information

Below is the link to the electronic supplementary material.

Supplementary file1 (XLSX 552 KB)

## Data Availability

All source data for this work (or generated in this study) are available upon reasonable request.
